# A Metamodel-Based Multi-Scale Reliability Analysis of FRP Truss Structures under Hybrid Uncertainties

**DOI:** 10.3390/ma17010029

**Published:** 2023-12-20

**Authors:** Desheng Zhao, Xiaoyi Zhou, Wenqing Wu

**Affiliations:** 1 Department of Bridge Engineering, School of Transportation, Southeast University, Nanjing 211189, China; 2020170683@mail.hfut.edu.cn (D.Z.); wuwenqing@seu.edu.cn (W.W.); 2 School of Civil Engineering, Hefei University of Technology, Hefei 230002, China

**Keywords:** fiber-reinforced polymer composite, multi-scale reliability analysis, hybrid random and interval uncertainties, metamodel, neural network, genetic algorithm, importance sampling

## Abstract

This study introduces a Radial Basis Function-Genetic Algorithm-Back Propagation-Importance Sampling (RBF-GA-BP-IS) algorithm for the multi-scale reliability analysis of Fiber-Reinforced Polymer (FRP) composite structures. The proposed method integrates the computationally powerful RBF neural network with GA, BP neural network and IS to efficiently calculate inner and outer optimization problems for reliability analysis with hybrid random and interval uncertainties. The investigation profoundly delves into incorporating both random and interval parameters in the reliability appraisal of FRP constructs, ensuring fluctuating parameters within designated boundaries are meticulously accounted for, thus augmenting analytic exactness. In application, the algorithm was subjected to diverse structural evaluations, including a seven-bar planar truss, an architectural space dome truss, and an intricate nonlinear truss bridge. Results demonstrate the algorithm’s exceptional performance in terms of model invocation counts and accurate failure probability estimation. Specifically, within the seven-bar planar truss evaluation, the algorithm exhibited a deviation of 0.08% from the established failure probability benchmark.

## 1. Introduction

FRP materials have received extensive attention in recent years due to their merits on strength, lightweight and corrosion resistance [1]. Industry sectors such as automotive, aerospace, marine, etc., have utilized these advantages to achieve significant breakthroughs in performance and design [2,3,4], and the construction sector was inspired by these achievements to use FRP materials to enhance structural performance, durability and safety of civil engineering structures [5,6]. For instance, FRP materials were used in truss bridges [7]. However, FRP materials have hierarchical structures consisting of multiple materials, and their complicated manufacturing processes inevitably introduce uncertainties in material properties, geometrical parameters, etc. In addition, these uncertainties may associate with parameters at micro- or macro-scales [8,9,10]. Uncertainties at multiscale parameters also introduce extra difficulties to quantify their influences on structural responses. In particular, these uncertainties may include both aleatory and epistemic variations in material properties. Hence, it is desirable to develop an efficient uncertainty quantification method to measure variations in structural performance of composite structures due to hybrid uncertainties in micro- and macro-scale parameters [11,12].

Notably, FRP’s multi-scale nature, especially its material phases across different scales, profoundly affects the safety and performance of the resulting structures [11,12]. In addition, variations in fiber volume fraction, material composition arising from manufacturing processes also significantly influence the design of FRP structures with clearly defined safety [8,13]. While traditional reliability analysis methods are relatively mature for conventional materials, their application to FRP structures often overlooks or overly simplifies micro-scale uncertainties [14]. Current methodologies remain limited, especially when addressing FRP composites’ microstructures and manufacturing defects [15]. To aptly address the challenges above, adopting multi-scale approaches is essential for the uncertainty analysis of FRP structures. These approaches include experimental studies, micro-mechanical modeling, and the combination of homogenization methods with stochastic finite element methods [16]. Uncertainty quantification techniques propagating uncertainties from the constituent material level to the structural level offer profound insights into the behavior of FRP structures [17]. Furthermore, given the high computational costs of conventional methods such as Monte Carlo simulations [18], efficient numerical approaches based on surrogate models have emerged as focal research areas [19], offering not only more accurate estimations for complex response surfaces, but also significantly reducing computational time and cost [20]. A central challenge in reliability analysis is to precisely evaluate the failure probability. This assessment encompasses many intricate factors, such as material properties, connection strategies, and loading conditions. The calculation of failure probability has thus attracted attention. For instance, Nassirian [21] delved into the finite element analysis of the tubular sections of FRP, Hassanzadeh [22] executed a comprehensive reliability assessment of specific FRP structures based on the ACI 440.1R-15 guidelines, and Hao [23] also systematically investigated structures potentially affected by explosive loads, exploring the reliability of concrete columns under such loads when reinforced with FRP.

Uncertainties have been widely divided into two categories: aleatory, inherent randomness in systems or processes, and epistemic, stemming from knowledge inadequacies [24,25,26,27]. Usually, the aleatory uncertainty is represented by random variable, while the epistemic uncertainty is described by interval variable. Unlike the reliability analysis with random variables only, one must assess the lower or upper bounds of failure probability when involving both random and interval uncertainties [28]. Conventional analysis methods, such as Monte Carlo simulations [29] and the Unified Uncertainty Analysis method based on the first-order reliability method [30] (abbreviated as UUA or FORM-UUA), each possess their unique strengths and limitations. For instance, while Monte Carlo simulations are ubiquitously employed, they necessitate many samples when addressing low-probability events, escalating computational costs. On the other hand, FORM-UUA may be constrained when confronted with multi-variable and highly non-linear scenarios. In the face of these high-dimensional, intricate, and intensely non-linear mixed uncertainty challenges, surrogate models progressively unveil their inherent value, emerging as the tool of choice for mixed uncertainty analysis [31]. They facilitate efficient uncertainty analysis within constrained computational resources.

Surrogate models have been introduced as efficient substitutes for the original Limit State Functions (LSF) [32], and a variety of meta-models, including quadratic response surfaces [33]], support vector machines [34,35,36], neural networks [37,38,39], Kriging methods [40], and other techniques, have been introduced. Notably, with their efficient learning capabilities, potent non-linear mapping abilities, and exceptional scalability, neural networks have gained significant attention [41,42]. The primary objective of constructing a meta-model is to ensure accurate evaluations of the actual performance function with a minimal sample size. Hence, active learning methods are widely adopted, iteratively optimizing the meta-model by selective sampling. Although active learning techniques based on Kriging, such as ALK-DIS [43], AK-SS [32], and AK-LS [36], have seen widespread application, ensuring model approximation accuracy remains a prominent challenge when dealing with rare events or high-dimensional issues. Typically, this necessitates the introduction of additional samples for meta-model updates. In this context, meta-heuristic algorithms, like genetic algorithms [39], have demonstrated their worth, adeptly pinpointing and augmenting critical samples, subsequently enhancing the meta-model’s construction efficiency and predictive capability. To further mitigate computational demands associated with meta-modeling [44], techniques like IS [43] and Subset Simulation [40] bolster the predictive accuracy and stability of meta-models, guaranteeing their reliability in intricate application scenarios for computational problems characterized by multiple failure region features and low failure probabilities, Cadini et al. [45] introduced a technique leveraging the K-means algorithm to cluster meta-model predicted failure points multiple times. While the resultant cluster count might exceed the actual identified failure regions by the meta-model, this method has been validated as efficient and rational.

This study aims to integrate variance reduction techniques with neural network meta-models, further optimizing the meta-model using genetic algorithms and offering an innovative approach for assessing the failure probability of FRP structures. Our preliminary work employed the Latin hypercube sampling method to generate samples, approximating the LSF using an RBF neural network. Building upon this foundation, the meta-model is optimized through genetic algorithms, continuously adjusting the sample set to elevate evaluation accuracy. An RBF-GA-BP-IS meta-model, combining the K-means algorithm, importance sampling techniques, and a two-layer BP neural network, is developed to conduct the multi-scale reliability analysis with considerations of both random and interval uncertainties.

## 2. Framework of a Metamodel-Based Multiscale Hybrid Reliability Analysis Method

### 2.1. Definition of Reliability Analysis Problem with Hybrid Random and Interval Uncertainties 

In the reliability analysis of FRP structures, defining LSF G to depict the structural response is a prerequisite. Various factors, such as displacement or buckling phenomena, can influence this structural response. We have chosen the maximum displacement as the primary criterion for the limit state function (LSF) due to its simplicity and computational efficiency. However, this study acknowledges that other failure modes, particularly global and local buckling, are critical in the reliability analysis of FRP structures and should be considered. In real-world applications, a structure may exhibit multiple failure modes, which can be interrelated and impact each other. 

The displacement-based limit state function, in the presence of both micro- and macro-scale parameters with random and interval uncertainties, can be expressed as follows:

(1) $$Z=G\left(x,y\right)$$

where x denotes the vector of random variables, while y signifies the vector of interval variables. Z is the response value of the function. 

With involving interval variables, it is necessary to find the optimal combination of interval variables $y_{M}$ or $y_{m}$ to maximize or minimize $G\left(x,y\right)$. In practice, it is paramount to determine the maximum of failure probability $P_{f}^{max}$, or the minimum of reliability index $\beta_{L}$. This following context thereby focuses on calculating $P_{f}^{max}$ or $\beta_{L}$. The lower bound of limit state function is determined first by using Genetic Algorithm to find the interval value $y_{m}$, and it will be used to finding the minimum of reliability index, $\beta_{L}$. The lower bound of limit state function $G\left(x,y_{m}\right)$ is defined as:

(2) $$G\left(x,y_{m}\right)\leq 0$$

Therefore, the FRP structure fails once $G\left(x,y_{m}\right)$ is less than zero, and $G\left(x,y_{m}\right)=0$ is the limit state between a failure domain $G\left(x,y_{m}\right)\leq 0$ and a safe domain $G\left(x,y_{m}\right)>0$.

### 2.2. A Metamodel Based Reliability Calculation Method

To determine $\beta_{L}$ defined by the limit state function in Equation (2), a novel approach based on integrating sampling techniques and metamodel generating methods is introduced. Sampling techniques include Monte Carlo Simulation (MCS), Importance Sampling (IS) and Genetic Algorithm (GA) are used, and metamodel methods include Radial Basis Function (RBF) and Back Propagation (BP) are included as well. MCS was employed first to facilitate comprehensive sampling, yielding a crucial set of sample points that laid the groundwork for subsequent metamodel construction. Drawing upon this dataset, the RBF neural network is used to establish a preliminary metamodel to capture the intrinsic behavioral characteristics of the system, striving for enhanced estimation accuracy, once the model met the predefined convergence criteria. IS is used to refine the sample set. Subsequently, to enhance precision of model, we opted for a dual-hidden layer BP neural network to construct a secondary metamodel. Throughout the research process, GA played a pivotal role, particularly in supplementing sample points and determining interval variable values, ensuring the efficiency and robustness of the overarching optimization process. The proposed RBF-GA-BP-IS strategy offers a novel and efficacious computational framework for the reliability analysis of FRP structures.

#### 2.2.1. Construction of Neural Network for Limit State Function

**Figure 1:**
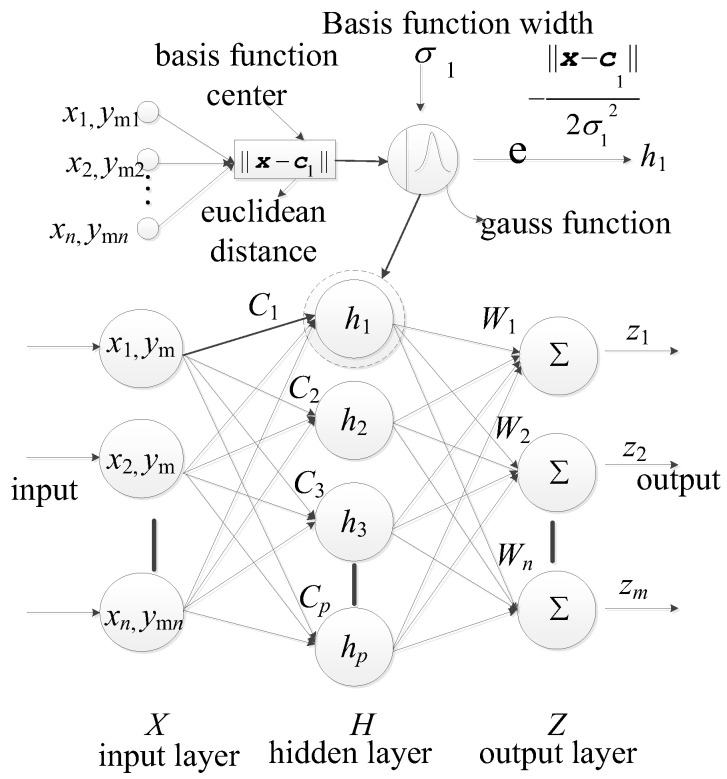
RBF network structure.

In the reliability analysis for FRP structures, an accurate limit state function is of the essence, as it directly pertains to estimating failure probabilities. Neural networks have been heralded as potent tools that simulate intricate nonlinear relationships [46]. Traditional mathematical models might be inadequate in capturing their inherent complexity for FRP structures. RBF (Figure 1) neural networks are tailor-made for local learning, especially during the preliminary stages [47]. For the RBF model, considering two inputs denoted as x and $y_{m}$, where $G\left(x,y_{m}\right)$ represents the output variable, the formula for the RBF Neural Network can be articulated as:

(3) $$G\left(x,y_{m}\right)=\sum_{i=1}^{N_{c}}w_{i}\phi \left(\left\|{\left[x,y_{m}\right]}^{T}-c_{i}\right\|\right)$$

where $w_{i}$ signifies the weights, ϕ denotes the selected radial basis function (in this study, Gaussian function), and $c_{i}$ stands as the center point, represented as a two-dimensional vector. $N_{c}$ stands for the number of center points.

**Figure 2:**
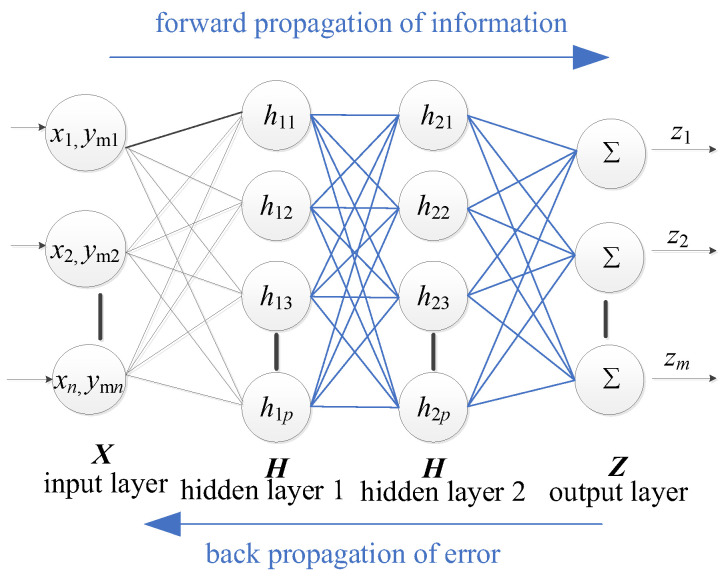
BP network structure.

However, as training samples increase, RBF neural networks may become overly complex and less efficient. To address this, we introduce the Bayesian Dual Hidden Layer BP neural network (Figure 2). While this network may exhibit slower training speeds, it provides a more precise modelling capability [48], which is particularly relevant for capturing the complex behaviors of FRP structures. Our methodology, therefore, begins with the RBF neural network for the initial training phases, especially when dealing with smaller datasets. As the dataset grows, we transition to the Bayesian Dual Hidden Layer BP neural network for more in-depth training. This hybrid approach amalgamates the swiftness of the RBF network with the precision of the Bayesian BP network. The formula for the Bayesian BP neural network can be presented as:

(4) $$G\left(x,y_{m}\right)=W_{2}\sigma \left(W_{1}{\left[x,y_{m}\right]}^{T}+b_{1}\right)+b_{2}$$

where $W_{1}$ and $W_{2}$ denote weight matrices, while $b_{1}$ and $b_{2}$ represent bias vectors. The function σ denotes the activation function.

It is imperative to recognize that both the RBF and the BP neural networks employ the mean squared error as their loss function. Within the RBF neural network, the MATLAB function “newrb” is integrated with GA to optimize the network architecture, mainly refining the width of the radial basis. In contrast, the BP neural network embraces a dual hidden-layer structure, permitting experimentation with diverse node combinations. The optimal node ensemble can be discerned by evaluating their performance on the loss function (with a recommended node count of 40 and 20). Furthermore, the BP neural network employs ReLU as the activation function for its hidden layers and designates “trainbr” as its training function.

#### 2.2.2. Structural Reliability Calculation with Importance Sampling 

Given the micro- and macro-scale parameters of FRP structures, the fusion of the Monte Carlo method with importance sampling offers a potent means to simulate and evaluate the repercussions of these random and interval variables. MCS has been used to estimate the failure probability [49] of FRP structures as follows:

(5) $${\hat{P}}_{f}=\frac{1}{N_{MCS}}\sum_{i=1}^{N_{MCS}}I\left[G\left(x_{i},y_{m}\right)\right]$$

where $I\left[\cdot \right]$ is the indicator function, and $N_{MCS}$ is the number of random samples.

**Figure 3:**
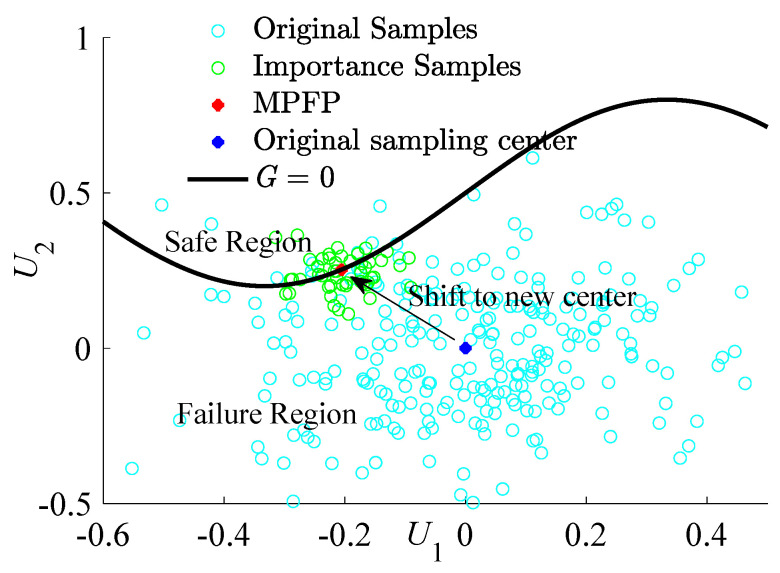
The process of adding important sampling points.

Importance sampling enables a more concentrated sampling of regions pivotal in failure analysis, thereby enhancing the overall simulation efficiency. Figure 3 illustrates the process of adding important sampling points, showing how this technique focuses on critical areas for failure analysis. With multiple vital areas to consider, each failure mode necessitates sampling at every design point. The expression for structural failure probability is as follows:

(6) $${\hat{P}}_{f}=\frac{1}{N_{i}}\sum_{i=1}^{M}\sum_{j=1}^{N_{i}}I\left[G\left(x_{j}^{\left(i\right)},y_{m}\right)\right]\frac{\prod_{l=1}^{N}f_{X_{i}}\left(x_{jl}^{\left(i\right)}\right)}{\sum_{k=1}^{M}\prod_{l=1}^{N}h_{V_{lk}}\left(x_{jl}^{\left(i\right)}\right)}$$

where $x_{j}^{\left(i\right)}$ represents the vector of random variables for the *j*-th random sample under the *i*-th failure mode; $f_{X_{i}}\left(x_{jl}^{\left(i\right)}\right)$ describes the probability density function for the *l*-th element in the vector of random variables $x_{j}^{\left(i\right)}$; $h_{V_{lk}}\left(x_{jl}^{\left(i\right)}\right)$ denotes the sampling density function for the *l*-th random variable in the *k*-th failure mode or importance sampling region, typically considered the normal distribution probability density function for the FRP structure; $N_{i}$ is the number of random samples under the *i*-th failure mode; M stands for the total number of failure modes or importance sampling regions, and N represents the length of the vector of random variables.

#### 2.2.3. Reliability Analysis with Multiscale and Hybrid Uncertainties

One must initially undertake an interval analysis based on metamodeling to ascertain the maximum failure probability. At this juncture, diverse y values are meticulously scrutinized. By leveraging genetic algorithms, we can pinpoint a specific $y_{m}$ such that, under this designated value, the metamodel manifests the zenith of failure probability. Our study progresses by iteratively adding sample points, refining our meta-model and enhancing its accuracy in capturing the nuances of interval uncertainties, ultimately leading to more precise determinations of maximum failure probabilities.

Subsequently, to adeptly capture the contours of the Maximum Failure Probability Points (MPFPs) [41], we transition to the phase of random variable analysis. During this phase, we employ a sampling technique rooted in genetic algorithms. This method offers precision in delineating the failure boundary and ensures a uniform distribution of samples. Consequently, a harmonious balance between accuracy and efficiency is achieved, enhancing our approximation of the MPFPs’ boundaries.

(7) $$\underset{U^{*}}{\min}\left\|u^{*}-C\right\|\;s.t.\left\{\begin{array}{c} \hat{G}\left(u^{*},y_{m}\right)=0\\ d_{min}>D\\ \left\|u^{*}-C\right\|<LR \end{array}\right.\;LR=\sqrt{\chi_{0.99}^{2}\left(N\right)}$$

(8) $$d_{min}=\underset{u^{*}}{\min}\left\{\left\|u^{*}-u_{k}\right\|\right\},\;k=1,2,\cdots ,N_{doe}$$

where, in the standard normal space U, considering N independent and identically distributed standard normal random variables, the value of LR is derived by utilizing the properties of the chi-squared distribution; C represents the sampling center coordinates of the sample, u is the Gaussian variable derived from the Nataf transformation of sample point x, and $d_{min}$ signifies the minimum distance from the new sample $u^{*}$ to the existing $N_{doe}$ sample points. $\hat{G}\left(u^{*},y_{m}\right)=0$ is the metamodel of the limit state function. The distance threshold D [50] can be selected as the maximum of the minimum Euclidean distances between the existing training samples, denoted as:

(9) $$D=\underset{}{\max}\left\{D_{i}|D_{i}=\underset{}{\min}\left\{\left\|X_{i}-X_{j}\right\|\right\},\;i\neq j,j=1,2,\cdots ,N_{doe}\right\}$$

As the number of new samples increases, D steadily decreases. For every incremental reduction in D, several new samples can be added. Historically, reducing D was a method to ensure feasible solutions to equations [42], though it was often time-consuming. Intending to augment the sample density near MPFPs and ensure a more uniform sample distribution, this paper introduces a constrained optimization problem. This problem is pivotal for approximating the LSF.

(10) $$\underset{u^{*}}{\min}\left(d_{min}\right)\;s.t.\left\{\begin{array}{c} \hat{G}\left(u^{*},y_{m}\right)=0\\ \left\|u^{*}-C\right\|\leq LR \end{array}\right.$$

During IS phases, it is paramount to recalibrate the sampling center of the samples. This adjustment becomes even more crucial when considering the complex nature of FRP structures. Due to the potential multiplicity of failure modes inherent to such structures, identifying individual failure domains and the associated Closest Failure Points (CFPs) within each becomes a daunting challenge.

**Figure 4:**
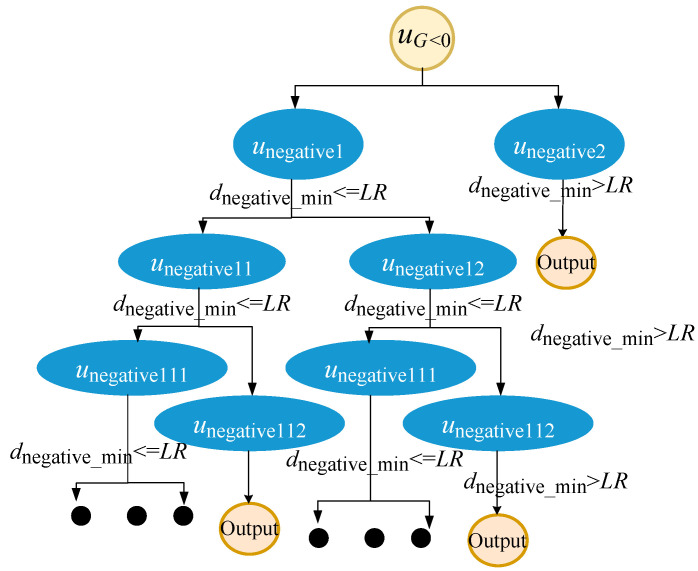
Find a binary tree diagram of multiple failure domains.

While algorithmic, this entire procedure can be visualized as the unfolding of a binary tree structure. At the heart of this tree lies the node $u_{\hat{G}}<0$, acting as the foundational root. Each segregation or bifurcation event yields two distinct nodes, $u_{negative\;I}$ and $u_{negative\;II}$, which emerge as the left and right offspring of $u_{\hat{G}}<0$. As the process advances, every subsequent split operation performed on $u_{negative\;I}$ and $u_{negative\;II}$ engenders further branching, resulting in additional child nodes. This recursive strategy, intricate as it sounds, beautifully mirrors a depth-first traversal on this binary tree. The entire sequence, from the initial sampling to the final categorization, can be vividly illustrated as depicted in Figure 4.

(11) $$d_{negative\_min}=\underset{}{\min}\left\{\left\|u_{i}-u_{CFPS}\right\|\right\},\;i=1,\;2,\cdots ,N_{negative}\;d_{negative\_min}\leq LR$$

where $d_{negative\_min}$ represents the minimum distance from the CFPs of the partitioned failure domain to the other samples in the failure domain $N_{negative}$, and $u_{CFPS}$ is the coordinate of the CFPs in the current partitioned failure domain.

### 2.3. Procedure of Numerical Implementation

**Figure 5:**
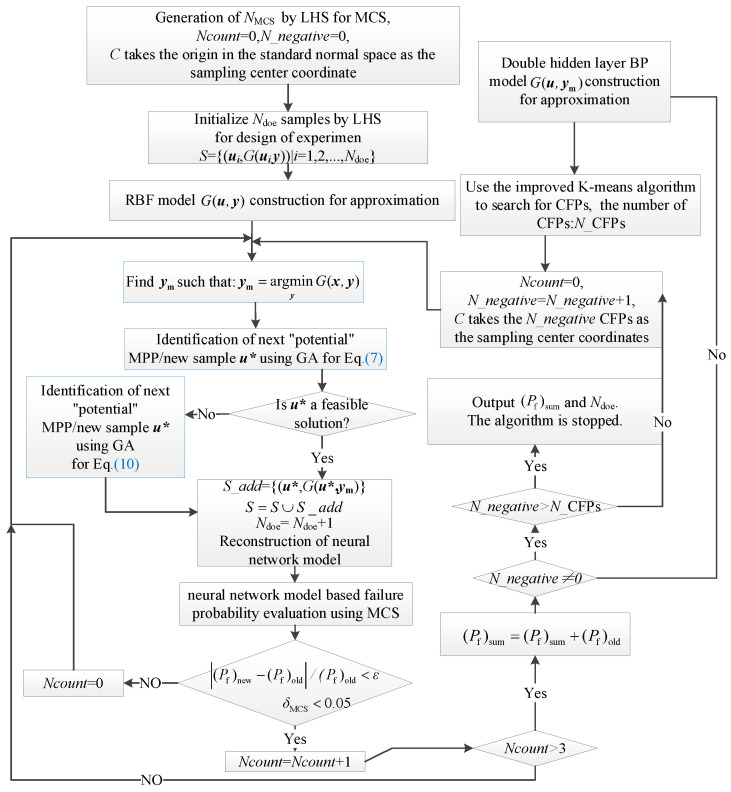
The flow chart of RBF-GA-BP-IS.

This section aims to elucidate the steps of the proposed RBF-GA-BP-IS reliability analysis method. As shown in Figure 5, a schematic representation of the algorithm is depicted in the flowchart provided. The algorithm encompasses two primary challenges: determining the interval variables $y_{m}$ and conducting a stochastic variable analysis. After each post metamodel reconstruction, interval variables are ascertained using a genetic algorithm. The stochastic variable analysis is bifurcated into two stages:

Stage 1 (RBF-GA): The RBF is a surrogate for intricate finite element model analyses. A preliminary reliability metamodel is established in conjunction with the GA sampling strategy.

Stage 2 (RBF-GA-BP-IS, RBF-GA-BP-IS^2^): During this stage, which can be further subdivided into RBF-GA-BP-IS and RBF-GA-BP-IS^2^, the distance-limited K-means algorithm is employed to pinpoint CFPs as sampling centers for importance sampling. Subsequently, a dual-hidden layer BP is combined with GA sampling to refine the metamodel. The distinction between RBF-GA-BP-IS and RBF-GA-BP-IS^2^ is the re-determination of CFPs in the latter, building upon the foundation of the former’s metamodel. The subsequent steps for both are analogous. When multiple failure modes manifest in stage 2, it is further segmented into sub-stages, for instance: stage 2Ⅰ-1, stage 2Ⅰ-2.

The efficacy of RBF-GA-BP-IS is exemplified through various case studies, all of which employ FRP materials, underscoring the method’s utility in the reliability analysis of FRP materials. Initially, an exemplar featuring a seven-bar planar truss was tested. Its selection was predicated on its explicit limit state function and its amenability to demonstrate the addition of sample points. Subsequently, the method was applied to a space dome truss structure. Despite its implicit limit state function expression, its structural simplicity and the brevity of computational time in ANSYS made it an apt choice. Both these cases are conducive to generating a plethora of sample points. Lastly, the method was executed on a high-dimensional nonlinear truss bridge, affirming the feasibility of RBF-GA-BP-IS for reliability analysis in FRP truss bridges.

## 3. Multiscale Finite Element Method for FRP Composite Structures

A pivotal challenge in the reliability of FRP is establishing a cohesive link between microscale parameters and the macroscopic structural response. The complexity of this analysis mandates the consideration of numerous uncertainties introduced during the manufacturing process [51]. Employing micromechanics and homogenization techniques becomes particularly crucial in multiscale finite element analyses. As highlighted in studies [52], leveraging these techniques facilitates the effective transfer of uncertainties from the microscale, culminating in a quantitative assessment of their impact on the macroscopic mechanical performance.

The Mori–Tanaka Homogenization Method [53,54,55], a seminal contribution to homogenization theories, overcomes the inherent shortcomings of the Rule of Mixtures (RM). While the RM is lauded for its straightforwardness, it frequently misjudges the transverse directional properties [56]. In contrast, the Mori–Tanaka method offers a refined technique by accounting for the mechanical interplay among distinct phases—a facet conspicuously missing in RM. Drawing from Eshelby’s strain-concentration tensor, this approach pivots on an ellipsoidal inclusion encapsulated within a matrix, furnishing a comprehensive perception of composite rigidity. Crucially, it amalgamates microscale and macroscale parameters, underscoring the profound influence of micro-parameters, such as material properties and microstructural configurations, on macroscopic mechanical attributes. This harmonious integration materializes through a meticulous homogenization procedure.

Mathematically, the overarching composite stiffness tensor, ${\bar{C}}_{MT}$, is articulated as follows:

(12) $${\bar{C}}_{MT}=C_{m}+V_{f}\left(C_{f}-C_{m}\right)A_{MT}$$

with

(13) $$A_{MT}=A_{Eshelby}{\left[V_{m}I+V_{f}A_{Eshelby}\right]}^{-1}$$

where $A_{Eshelby}={\left[I+E_{t}S\left(C_{f}+C_{m}\right)\right]}^{-1}$ is Eshelby’s strain-concentration tensor, I represent the identity tensor. $E_{t}$ is the Eshelby tensor, contingent solely upon the inclusion’s aspect ratio and the matrix’s elastic constants. Herein, S and C signify the compliance and stiffness tensors, respectively.

The Mori–Tanaka tensor, delineated for unidirectional fiber-reinforced composites (comprising transversely isotropic fibers and an isotropic matrix), is depicted as:

(14) $$A^{\mathrm{MT}}=\left[\begin{array}{llllll} A_{11} & A_{12} & A_{13} & 0 & 0 & 0\\ A_{21} & A_{22} & A_{23} & 0 & 0 & 0\\ A_{31} & A_{32} & A_{33} & 0 & 0 & 0\\ 0 & 0 & 0 & A_{44} & 0 & 0\\ 0 & 0 & 0 & 0 & A_{55} & 0\\ 0 & 0 & 0 & 0 & 0 & A_{66} \end{array}\right]$$

The non-zero elements of this tensor emanate from the intrinsic properties of the materials involved, namely, the elastic modulus *E*, Poisson ratio v, and shear modulus *G*.

Subsequently, Equation (15) detail these elements.

(15) $$\begin{array}{l} A_{11}=\frac{E^{m}}{E_{11}^{f}}\left[1+\frac{v^{m}\left(v^{m}-v_{12}^{f}\right)}{\left(1+v^{m}\right)\left(1-v^{m}\right)}\right]\\ A_{12}=A_{13}=\frac{E^{m}}{E_{22}^{f}}\frac{v^{m}\left(1-v_{23}^{f}\right)}{2\left(1+v^{m}\right)\left(1-v^{m}\right)}-\frac{E^{m}}{E_{11}^{f}}\frac{v_{12}^{f}}{\left(1+v^{m}\right)\left(1-v^{m}\right)}+\frac{v^{m}}{2\left(1-v^{m}\right)}\\ A_{21}=A_{31}=\frac{E^{m}}{E_{11}^{f}}\frac{v^{m}-v_{12}^{f}}{2\left(1+v^{m}\right)\left(1-v^{m}\right)}\\ A_{22}=A_{33}=\frac{E^{m}}{E_{22}^{f}}\frac{\left(v_{23}^{f}-3\right)}{8\left(v^{m}-1\right)\left(v^{m}+1\right)}+\frac{E^{m}}{E_{11}^{f}}\frac{v_{12}^{f}v^{m}}{2\left(v^{m}-1\right)\left(v^{m}+1\right)}+\frac{\left(v^{m}+1\right)\left(4v^{m}-5\right)}{8\left(v^{m}-1\right)\left(v^{m}+1\right)}\\ A_{32}=A_{23}=\frac{E^{m}}{E_{22}^{f}}\frac{\left(3v_{23}^{f}-1\right)}{8\left(v^{m}-1\right)\left(v^{m}+1\right)}+\frac{E^{m}}{E_{11}^{f}}\frac{v_{12}^{f}v^{m}}{2\left(v^{m}-1\right)\left(v^{m}+1\right)}+\frac{\left(v^{m}+1\right)\left(1-4v^{m}\right)}{8\left(v^{m}-1\right)\left(v^{m}+1\right)}\\ A_{44}=\frac{G^{m}}{G_{23}^{f}}\frac{1}{4\left(1-v^{m}\right)}+\frac{\left(3-4v^{m}\right)}{4\left(1-v^{m}\right)}\\ A_{55}=A_{66}=\frac{G^{m}+G_{12}^{f}}{2G_{12}^{f}} \end{array}$$

Consequently, formulas for the five independent equivalent elastic parameters of isotropic fiber-reinforced composites are derived Equation (12).

## 4. Numerical Examples

### 4.1. A Seven-Bar Planar FRP Truss Structure 

**Figure 6:**
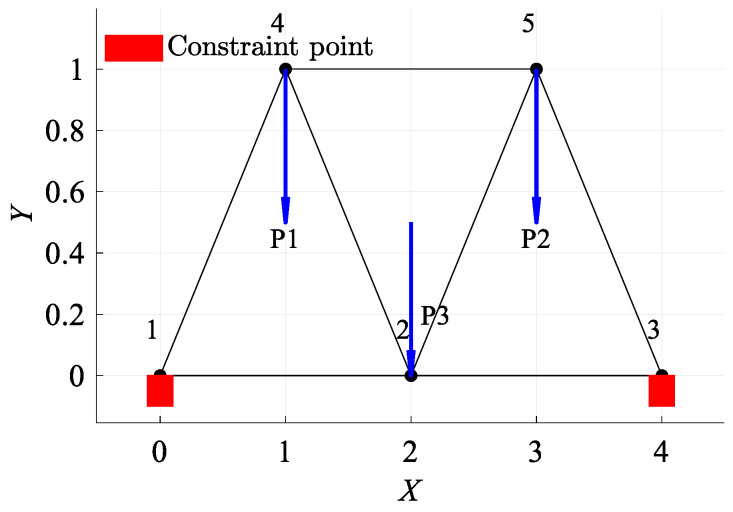
The schematic view of planar 7-bar structure /m.

This section introduces the application of the RBF-GA-BP-IS methodology in the reliability analysis of a seven-bar FRP planar truss, as shown in Figure 6. It is used to demonstrate applicability of the proposed method by comparing with the conventional method based on the Monte Carlo simulation. Middle span deflection has been considered, and the limit state function is defined as [57,58]:

(16) $$G\left(x,y\right)=d_{max}-d\left(x,y\right)=d_{max}-\frac{H}{EA}=d_{max}-\frac{\left(1+\sqrt{2}\right)\left(2P_{1}+P_{2}+P_{3}\right)}{EA}$$

(17) $$E=E_{f}V_{f}+E_{m}V_{m}$$

where neglecting the self-weight of the members, nodes 4, 5, and 2 are subjected to concentrated loads represented by $P_{1}$, $P_{2}$, and $P_{3}$, respectively. The term $d_{max}$ designates the maximum permissible displacement, set at 40 mm. E stands for Young’s modulus, where $E_{f}$ and $E_{m}$ represent the elastic moduli of the fiber and matrix, respectively. A denotes the cross-sectional area with a value of $0.1\;m^{2}$, and $d\left(x,y\right)$ is the analytical expression for mid-span displacement.

The truss is fabricated from GFRP and contemplates various parameter combinations. As listed in Table 1, specific parameters, like the fiber and matrix elastic moduli, are perceived as random variables. Meanwhile, others, such as $V_{f}$ and $P_{3}$, are regarded as interval variables. 

**Figure 7:**
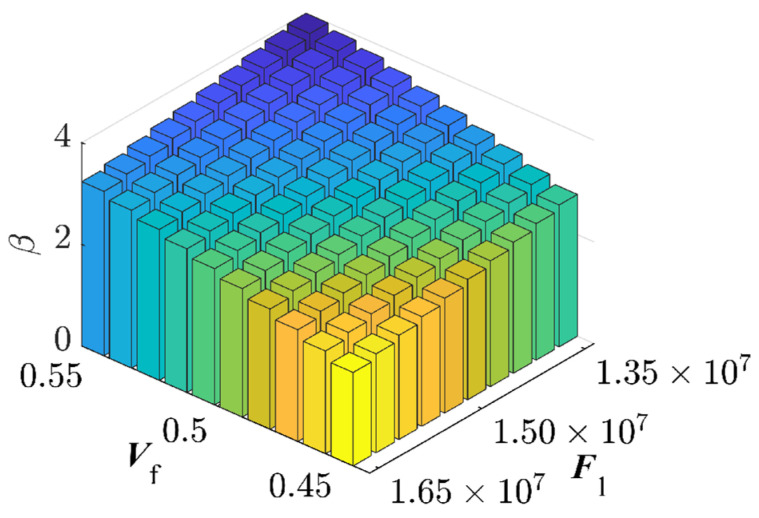
Reliability Indices for Seven-Bar Truss Structure.

**Figure 8:**
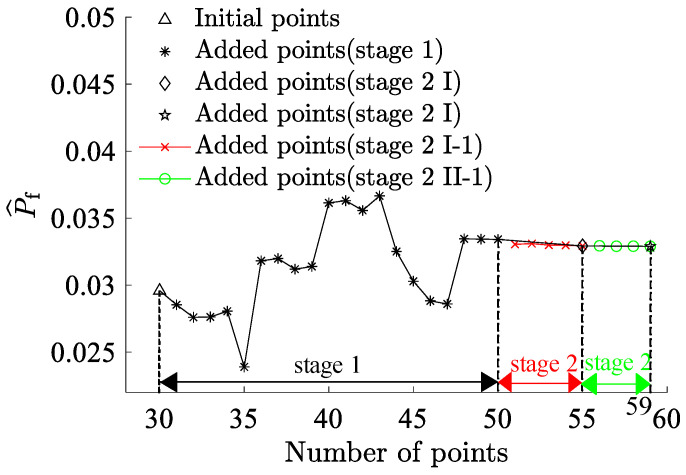
Failure Probability Convergence for Seven-Bar Truss.

By partitioning the two interval variables, $V_{f}$ and $P_{3}$, into ten equal segments and combining them pairwise, a total of 100 interval variable combinations were formed. Each set randomly selected $5\times 10^{10}$ samples (Figure 7). The combination exhibits the highest failure probability consists with $V_{f}=45\%$ and $P_{3}=1.65\times 10^{7}\;N$, resulting in a benchmark failure probability of $P_{f}=3.29\%$, and the reliability index, β=1.8394. This reaffirms that genetic algorithms adeptly identify combinations with the highest failure probabilities. The RBF-GA-(BP-IS)^2^ method deviates from the benchmark failure probability by a mere 0.079%, making the RBF-GA-BP-IS approach superior in precision to other methods. Figure 8 illustrates refining the accuracy of failure probability ${\hat{P}}_{f}$ by increasing the number of sample points. Each phase improves the estimation of ${\hat{P}}_{f}$ by incorporating additional sample points; initially, in Stage 1, there is considerable fluctuation, but in subsequent stages, the ${\hat{P}}_{f}$ progressively stabilizes. Table 2 infers that RBF-GA-BP-IS and RBF-GA-BP-IS^2^ demonstrate significant advantages in model performance assessment. Both methods exhibit enhanced efficiency regarding model invocation counts, especially when contrasted with the computation-intensive MCS method. More critically, both excel in estimating failure probability with remarkable accuracy.

**Figure 9:**
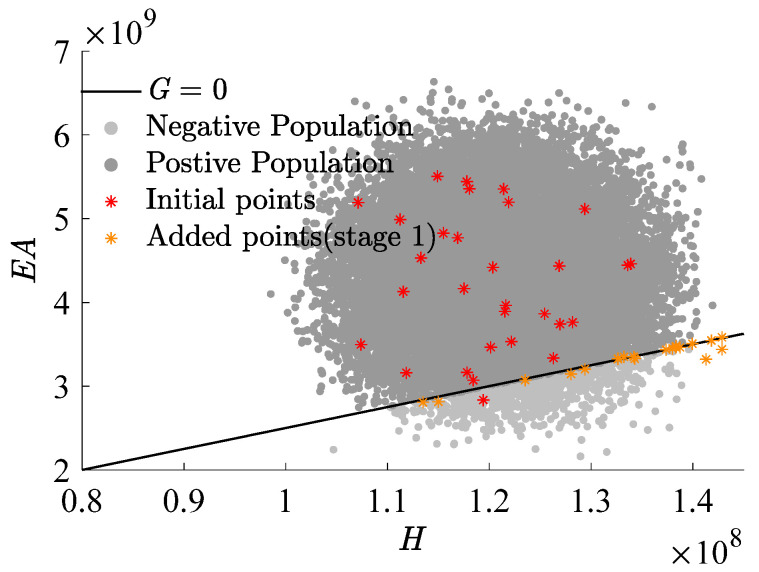
Add points process (stage 1).

**Figure 10:**
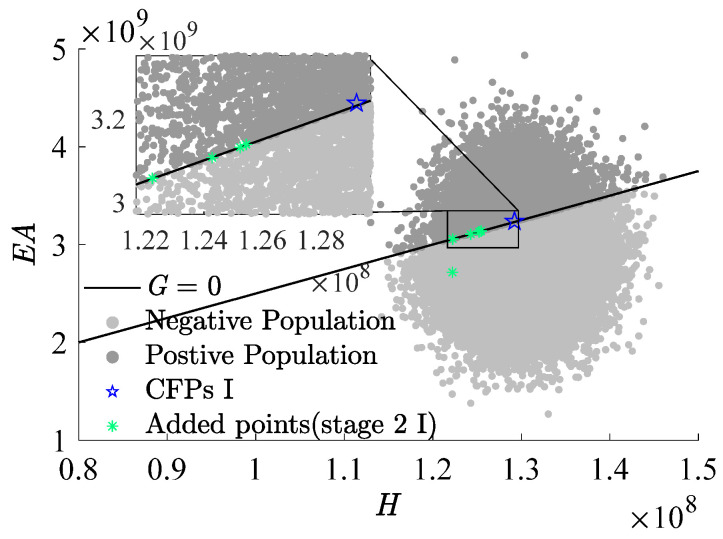
Add points process (stage 2 I).

**Figure 11:**
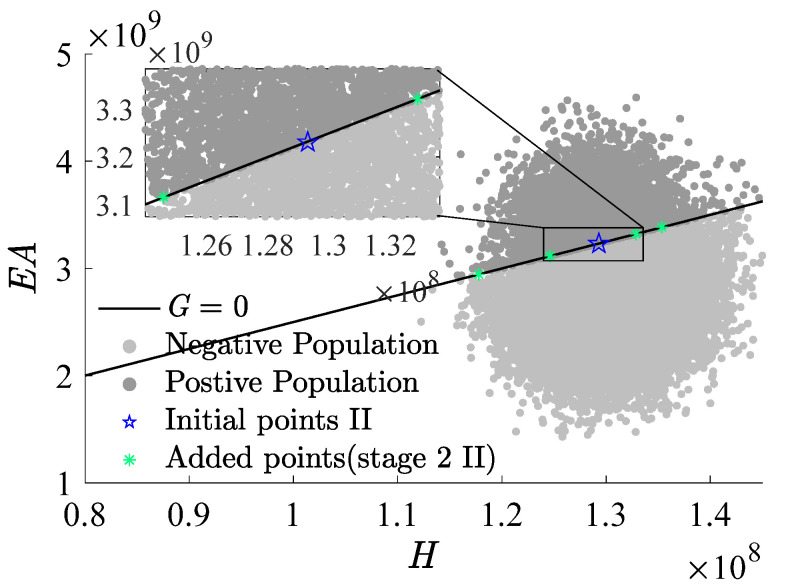
Add points process (stage 2 II).

Figure 9, Figure 10 and Figure 11, which use H as the x-axis and EA as the y-axis, demonstrate the iterative addition of sample points and their effects. Figure 10 and Figure 11 reveal that via post-important sampling, new sample points are more concentrated in critical regions that significantly influence simulations. Within the sample space, “Initial points” manifest a dispersed distribution, laying a solid foundation for constructing the first-stage metamodel. Subsequent sample points are closely adjacent to the actual limit state interface, especially during the second stage’s CFPs (encompassing stage 2 I and stage 2 II). When dimensions are relatively low, the RBF-GA-BP-IS strategy already procures a fairly accurate failure probability, attesting to the algorithm’s precision and robustness.

### 4.2. A Space Dome Truss Structure

**Figure 12:**
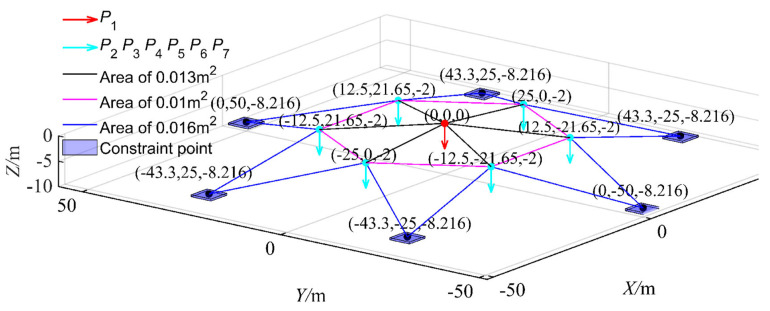
Schematic view of elements, applied loads and geometry of space truss.

As shown in Figure 12, this study offers a meticulous analysis of the response of a spatial truss composed of 24 members under seven distinct load scenarios. This instance delves into a high-dimensional truss structure problem, particularly emphasizing its implicit function limit state. This limit state is delineated based on the maximum displacement of nodes in the z-direction under specific loads. LSF is defined as [59,60,61]:

(18) $$G=0.0092-\left|\Delta_{P_{1}}^{z}\right|$$

where $\left|\Delta_{P_{1}}^{z}\right|$ signifies the maximum displacement in the z-direction, the problem encompasses ten random variables, including loads $P_{1}-P_{7}$, fiber volume fraction $V_{f}$, elastic moduli $E_{f}$ and $E_{m}$ Their statistical characteristics are detailed in Table 3.

**Figure 13:**
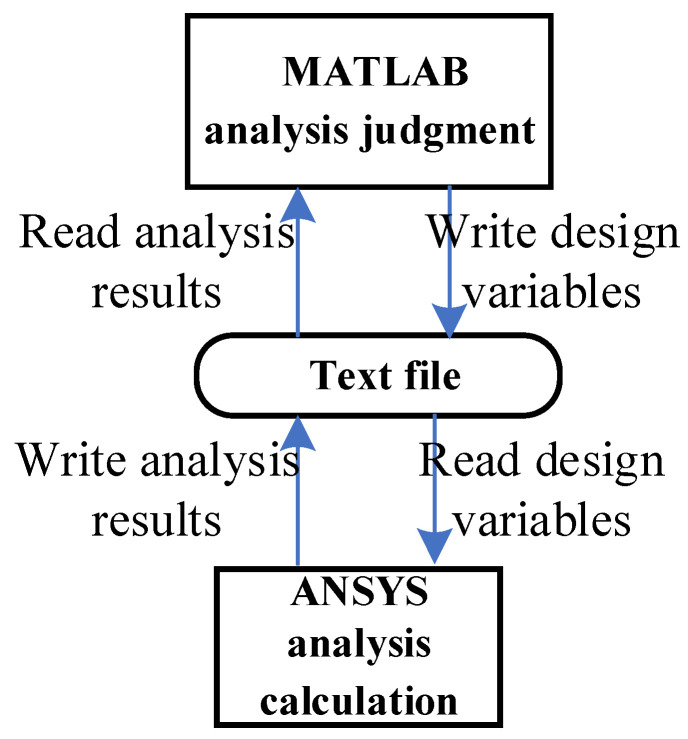
Data transmission direction between MATLAB and ANSYS.

**Figure 14:**
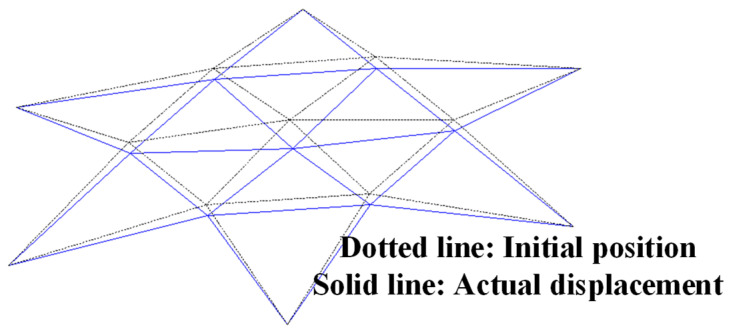
Schematic view of deformation shape based on finite element model for space truss.

For the spatial truss structure depicted, relying on the performance above function, the maximum displacement constraint is 0.0092 m. This displacement is derived from ten random variables. To further scrutinize this displacement, the finite element analysis integrating MATLAB and ANSYS is used to conduct a parametric analysis model. Notably, leveraging MATLAB and ANSYS’s prowess in handling text files, efficient data exchange between the software suites is achieved as elaborated in Figure 13. The schematic representation of nodal displacements under applied loads is showcased in Figure 14, where $V_{f}$ is at its minimum, $P_{1}$ at its zenith, and other random variables at their mean values, resulting in a maximum z-direction displacement of $\left|\Delta_{P_{1}}^{z}\right|=7.27\times 10^{-3}\;m$.

**Figure 15:**
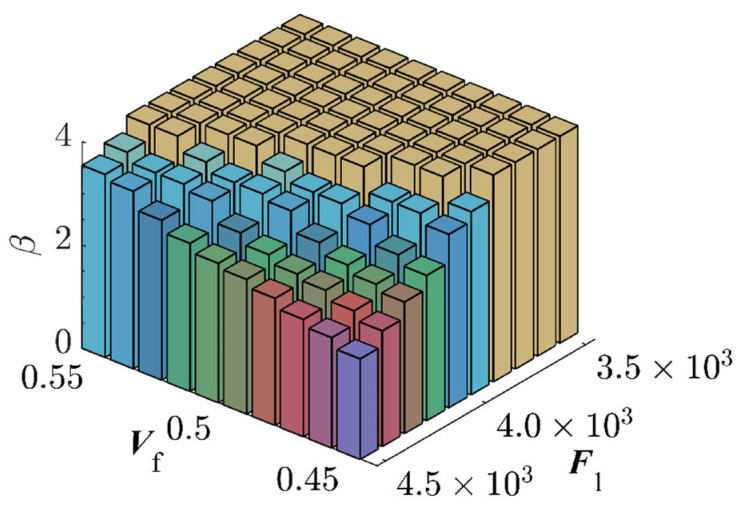
Reliability Indices for Space Dome Truss.

**Figure 16:**
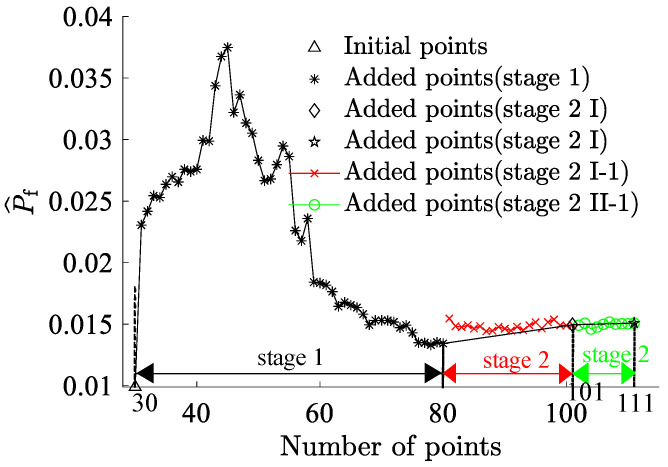
Failure Probability Convergence for Space Dome Truss.

MCS was employed for algorithmic validation in this research endeavor. The fiber volume fraction $V_{f}$ and the load $P_{1}$ were subdivided into ten segments, and their combinations yielded 100 interval variables. Each combination underwent $1\times 10^{6}$ sampling iterations. The simulation results are graphically represented in Figure 15. The combination exhibiting the highest failure probability was $V_{f}=45\%$ and $P_{1}=1.65\times 10^{7}N$, with a failure probability reaching 1.51%. Due to simulation time constraints in ANSYS, MCS with a large sample size is challenging. Thus, the Bayesian Backpropagation Neural Network [62] has been used to approximate the samples with the highest failure probability, obtaining a benchmark failure probability of 1.51% for this study. Table 4 presents the comparative outcomes of various methodologies. It is evident that the RBF-GA-BP-IS method’s deviation from the Bayesian Neural Network is only 1%, and the RBF-GA-BP-IS^2^ method’s deviation is even more negligible at 0.14%. The proposed RBF-GA-(BP-IS)^2^ algorithm demonstrates remarkable efficacy and accuracy. Figure 16 demonstrates that with the increase in sample points, the failure probability converges and aligns closely with the established benchmark failure probability. Despite the limitation in the number of invocations, the approach consistently yields a failure probability comparable to that of the Monte Carlo Simulation. It sustains a negligible error margin relative to the Bayesian Neural Network. This provides an efficient and reliable methodology for assessing the reliability of materials and structures.

### 4.3. A GFRP Truss Bridge

With the rapid progression of composite material technology, GFRP truss bridges [63] have found extensive applications in contemporary engineering projects. Due to their unique material attributes and structural features, an accurate and efficient assessment of their reliability becomes imperative. Given the complexity of these bridges, the analytical derivation of their structural response is challenging. Hence, an interactive computation method between MATLAB and ANSYS has been employed, as shown in Figure 13.

#### 4.3.1. Finite Element Analysis and Parametric Analysis of the GFRP Truss Bridge

**Figure 17:**
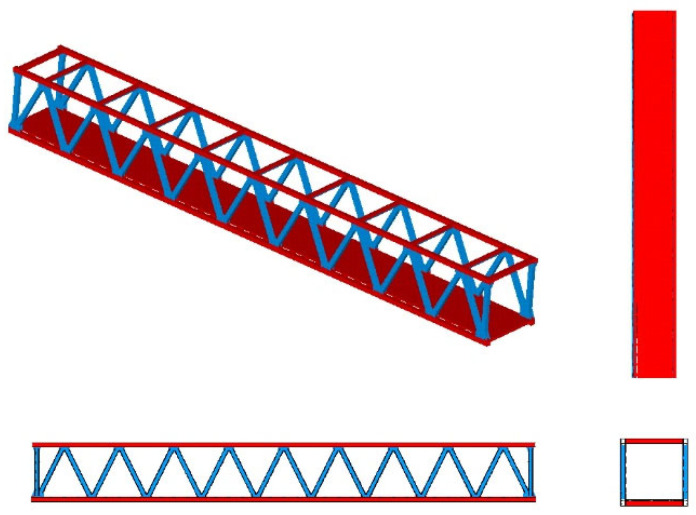
Finite element model.

A GFRP truss bridge with a span length of 36 m and a width of 8 m, as shown in Figure 17, has been studied [57]. The top and bottom chords incorporate hollow rectangular sections measuring 300 × 300 × 16 mm, while diagonal members and terminal crossbeams adopt a 300 × 300 × 12 mm hollow rectangular section. Horizontal connections utilize an I-shaped section of 300 × 200 × 15 mm. Each structural member comprises three layers across the sectional depth. The first and third layers are 0/90° balanced layup with a fiber content of 40% and a thickness of 0.4 mm. The intermediate layer is a fabric layer with a fiber content of 55%, where fibers are aligned longitudinally. The microscopic parameters are further detailed in Table 5.

**Figure 18:**
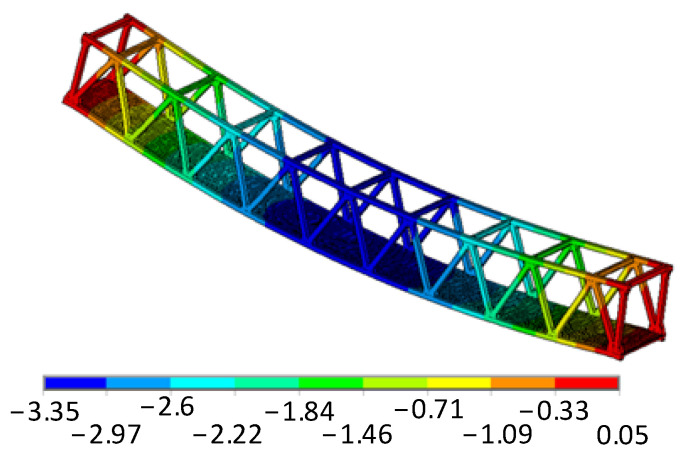
Deformed model with vertical displacement /cm.

This study particularly emphasizes the impacts of multi-scale and multi-source uncertainties on bridge reliability and the construction of metamodels. Relying on literature references [57,64], we delve into the microscopic mechanical properties of the glass fiber and matrix. As illustrated, considering all random and interval variables under imposed load and self-weight at their mean or central values (Table 6), the maximum vertical displacement is determined to be 0.0335 m(Figure 18).

#### 4.3.2. Classification and Characteristics of Uncertainty Parameters

This research delves deeply into uncertainties across different length scales. These uncertainties can be broadly categorized into two domains: macroscopic and microscopic. At the macroscopic scale, uncertainties mainly pertain to the overall structural characteristics, such as section dimensions and external loads. Given that abundant experimental data and historical records typically support these parameters, they are considered random variables. For instance, the density of fiberglass and pedestrian loads are random variables based on a normal distribution [65]. Microscopic scale uncertainties pertain to intrinsic material properties [66], such as elastic moduli of fibers and matrix, fiber volume fraction, and Poisson’s ratio. Given the paucity of data on these microscopic parameters, they are treated as interval variables, providing a probable range. Though GFRP truss bridge structures are not ubiquitous in real-world applications, their unique attributes and potential applicability make them a focal point of research. Most existing studies concentrate on their macroscopic mechanical properties [67], while investigations into their microscopic attributes still need clarification.

As listed in Table 6, statistics of uncertainty parameters are elaborately detailed [55,57]. The reliability of structural systems always corresponds to performance or limit state functions. To circumvent superfluous computations, only those uncertainty parameters with significant impact are considered, leading to the following LSF:

(19) $$G\left(x,y\right)=d_{max}-d\left(\rho ,\;q,\;E_{f},\;E_{m},\;V_{f},\;\mu_{f},\;\mu_{m}\right)$$

where $d_{max}$ represents the limit value of bridge displacement, which is set at the stipulated deflection of truss bridges, L/800. The material density ρ is calculated based on a permissible deviation of 7% in unit area mass as stipulated in reference [57] and converted into a variation coefficient of 0.023 using the 3σ rule. The volume fraction of fibers $V_{f}$ is governed by the regulations in [57], with an allowable deviation of ±3% for glass fiber content. For the remaining parameters, there is limited experimental data, so assumptions are made based on the data shown in the table.

#### 4.3.3. Reliability Analysis of GFRP Truss Bridge

In this study, the Bayesian Regularization Neural Network has been used to fit 3000 random sample points to ascertain the limit state function. Considering the volume fraction of fibers $V_{f}$ and the fiber Poisson’s ratio $\mu_{f}$ and matrix Poisson’s ratio $\mu_{m}$ as interval variables, we divided them into ten equal-length intervals, resulting in 1000 sets of interval variables. We randomly selected $1\times 10^{6}$ samples for each set and inputted them into the Bayesian Regularization Neural Network for computation. Post-analysis revealed that the combination with the highest failure probability was $V_{f}=52\%$, $\mu_{f}=0.22$ and $\mu_{m}=0.32$. This aligns closely with the interval variables derived from the RBF-GA-BP-IS^2^ metamodel method, as listed in Table 7.

**Figure 19:**
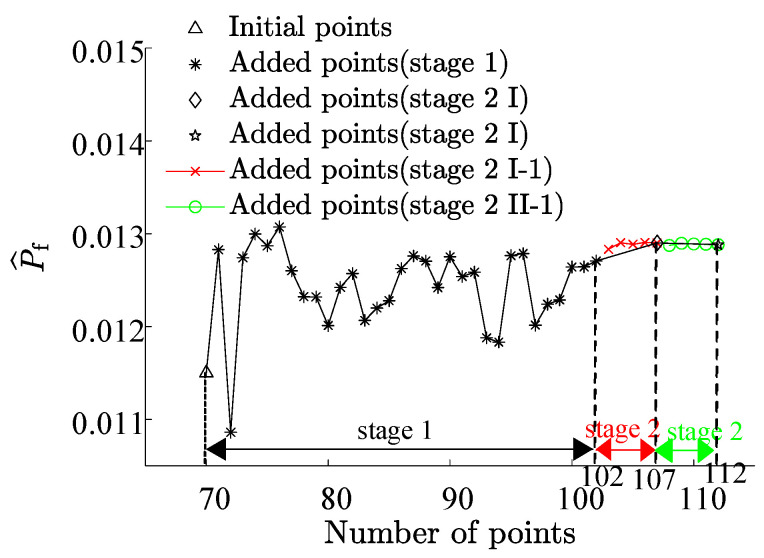
Failure Probability Convergence for GFRP Truss Bridge.

Under the combination with the highest failure probability, we revisited the Bayesian Regularization Neural Network, this time fitting 3000 sample points and then using $1\times 10^{9}$ sample points to determine the failure probability. Based on this, we regarded the failure probability obtained from the Bayesian Regularization Neural Network as the benchmark failure probability. As shown in Table 8, the RBF-GA-BP-IS method’s failure probability was close to the benchmark, with a marginal error of 0.06. As shown in Figure 19, with the increment in initial sample points, the failure probability ${\hat{P}}_{f}$ derived from our research methodology gradually stabilizes, converging closer to the comparative solution. It is imperative to note that due to the model’s intricacy, the Bayesian Regularization Neural Network underwent only 3000 simulations in ANSYS. Hence, there is a constraint on the precision of the failure probability, allowing us only to approximate the accuracy of truss bridges under RBF-GA-BP-IS^2^.

In the current study, we recognize that due to considerations of computational efficiency and the curse of dimensionality, our model does not directly account for fabrication defects [68] that may occur, such as imperfections in joints or struts. While these defects could significantly impact the structural performance and reliability, at this stage, we have chosen to focus on our current model’s core functionalities and efficiency. Future work will further consider these additional factors to enhance the model’s comprehensiveness and accuracy.

## 5. Conclusions

This study introduces a new multi-scale reliability analysis method based on a metamodel, which combines the RBF neural network, GA sampling, BP neural network, and IS, for FRP truss structures. It merges the quick processing of the RBF network with the accuracy of the Bayesian BP network, providing a refined homogenization method for composite materials and structures. While applying the Mori–Tanaka method in this research allows for consideration of the mechanical interaction among distinct phases in the composite material, the proposed method provides a comprehensive understanding of composite structures comparing with traditional methods, such as Rule of Mixture. In addition, the present study enables to simultaneously consider both random and interval variables in the reliability analysis of FRP structures. This inclusive approach ensures that fluctuating parameters within a specific range are considered, enhancing the accuracy of the analysis.

The proposed method has been successfully applied to multiple case studies, including a seven-bar planar truss, a space dome truss structure, and a complex nonlinear truss bridge. The results attest to the algorithm’s superior performance in model invocation counts and precise failure probability estimation. For instance, in the seven-bar planar truss case, the RBF-GA-BP-IS approach showed a minimal error of 0.002%, indicating its precision. Applying the RBF-GA-BP-IS method to the analysis of FRP truss bridges represents a significant step forward. This case study highlights the algorithm’s practical utility and contributes to the growing body of research on the reliability analysis of such structures. Worth noting is that the algorithm’s failure probability was close to the benchmark, with a minor error of 0.06%, affirming its reliability.

In conclusion, the RBF-GA-BP-IS algorithm presented in this study is valuable to the toolkit for multi-scale reliability analysis of FRP materials and structures. Its ability to handle both random and interval variables effectively and its successful application to various case studies marks it as a helpful tool for future research in this field.

## Figures and Tables

**Table 1:** Results of different methods (stage RBF-PSO).

Variables	Symbol	Unit	Mean Value or Central Value	Coefficient of Variation or Interval Radius	Distribution Type
Fiber volume fraction	$V_{f}$	%	50	5	Interval
Elastic modulus of fibers	$E_{f}$	GPa	82.45	0.1	Normal
Elastic modulus of matrix	$E_{m}$	GPa	3.45	0.1	Normal
Point load	$P_{3}$	N	$1.5\times 10^{7}$	$1.5\times 10^{6}$	Interval
Point load	$P_{1}$	N	$10^{7}$	0.1	Normal
Point load	$P_{2}$	N	$10^{7}$	0.1	Normal

**Table 2:** Performance Comparison for Seven-Bar Planar FRP Truss.

Method	$N_{call}$	${\hat{P}}_{f}\;\left(\%\right)$	${\Delta \hat{P}}_{f_{1}}\left(\%\right)$	${\Delta \hat{P}}_{f_{2}}\left(\%\right)$	${\Delta \hat{P}}_{f_{3}}\left(\%\right)$
MCS	$5\times 10^{12}$	3.29	-	-	-
RBF-GA	30+20	3.34	1.48	-	-
IS	$5\times 10^{6}$	3.29	-	-	-
RBF-GA-BP-IS	30+20+5	3.29	0.002	0.002	-
IS^2^	$5\times 10^{6}$	3.29	-	-	-
RBF-GA-BP-IS^2^	30+20+5+4	3.29	0.079	-	0.028

Note: IS refers to the critical sampling conducted at the center of CFPs under stage 2 I, where the probability of failure is determined by inserting it into the limit state function. IS^2^ indicates the critical sampling done at the center of CFPs under stage 2 II, and the failure probability is computed similarly. ${\Delta \hat{P}}_{f_{1}}$ represents the percentage error in the probability of failure compared to MCS. ${\Delta \hat{P}}_{f_{2}}$ represents the percentage error in the probability of failure compared to IS. ${\Delta \hat{P}}_{f_{3}}$ represents the percentage error in the probability of failure when compared to IS^2^.

**Table 3:** Statistical properties of random variables for space truss structure.

Variables	Symbol	Unit	Mean Value or Central Value	Coefficient of Variation or Interval Radius	Distribution Type
Fibre volume fraction	$V_{f}$	%	50	5	Interval
Elastic modulus of fibres	$E_{f}$	GPa	82.45	0.1	Normal
Elastic modulus of matrix	$E_{m}$	GPa	3.45	0.1	Normal
Point load	$P_{1}$	N	4000	500	Interval
Point load	$P_{2}$~$P_{7}$	N	3000	0.2	Normal

**Table 4:** Performance Comparison for Space Dome Truss.

Method	$N_{call}$	${\hat{P}}_{f}\left(\%\right)$	${\Delta \hat{P}}_{f}\left(\%\right)$
MCS	$1\times 10^{6}$	1.50	-
Bayesian regularization Neural Network	$1\times 10^{6}$	1.51	-
RBF-GA	30 + 50	1.34	11.06
RBF-GA-BP-IS	30 + 50 + 21	1.49	1
RBF-GA-BP-IS^2^	30 + 50 + 21 + 10	1.51	0.14

Note: ${\Delta \hat{P}}_{f}$ represents the percentage error in the probability of failure compared to Bayesian regularization Neural Network.

**Table 5:** Macro and micro mechanical properties of GFRP truss bridge.

Micro parameters		$E\;\left(GPa\right)$				μ	
	Fibre	82.45				0.20	
	Matrix	3.45				0.35	
Macro parameters		$V_{f}\;\left(\%\right)$	$\theta \;\left({}^{\circ}\right)$	$E_{11}\;\left(GPa\right)$	$E_{22}\;\left(GPa\right)$	$G_{12}\;\left(GPa\right)$	$\mu_{12}$
	Fabric layer	55	0	46.92	10.16	3.94	0.26
	Yarn layer	40	0/90	21.40	21.40	2.78	0.1

**Table 6:** Statistics of uncertain parameters of the GFRP truss bridge.

Parameters		Symbol	Mean/Central Value	Coefficient of Variation/Interval Radius	Distribution Type
Scale	Name				
Macro	Density	ρ	1800	0.023	Normal
	Applied load	q	2200	0.10	Normal
Micro	Elastic modulus of fibres	$E_{f}$	82.45	0.1	Normal
	Elastic modulus of matrix	$E_{m}$	3.45	0.1	Normal
	Fibre volume fraction	$V_{f}$	55	3	Interval
	Poisson Ratio of Fibres	$\mu_{f}$	0.2	0.02	Interval
	Poisson Ratio of Matrix	$\mu_{m}$	0.35	0.035	Interval

**Table 7:** Interval analysis results of reliability indices with displacement-based limit state functions.

Interval Variables	$V_{f}$	$\mu_{f}$	$\mu_{m}$
Results	0.52000	0.21985	0.31503
Upper/Lower limit	Lower	Upper	Lower

**Table 8:** Performance Comparison for GFRP Truss Bridge.

Method	$N_{call}$	${\hat{P}}_{f}$	$\Delta {\hat{P}}_{f}$
Bayesian regularization Neural Network	$3\times 10^{3}$	1.29	-
RBF-GA	70 + 32	1.27	1.44
RBF-GA-BP-IS	70 + 32 + 5	1.29	0.06
RBF-GA-BP-IS^2^	70 + 32 + 5 + 5	1.29	0.08

Note: $\Delta {\hat{P}}_{f}$ represents the percentage error in the probability of failure compared to Bayesian regularization Neural Network.

## Data Availability

Data will be made available on request.
